# Reproductive ecology of interior least tern and piping plover in relation to Platte river hydrology and sandbar dynamics: Response to the letter to the editor

**DOI:** 10.1002/ece3.4097

**Published:** 2018-05-02

**Authors:** Jason M. Farnsworth, David M. Baasch, Chadwin Smith, Kevin L. Werbylo

**Affiliations:** ^1^ Platte River Recovery Implementation Program Kearney Nebraska

## Abstract

This is a response to the Alexander, Jorgensen, and Bomberger‐Brown (Ecology and Evolution, XX, 2018, XX; hereafter, AJB) Letter to the Editor critiquing Farnsworth et al. (Ecology and Evolution, 7, 2017, 3579; hereafter, our study), which investigates the reproductive ecology of interior least terns and piping plover in relation to Platte River hydrology and sandbar dynamics. Herein, we address each of AJBs’ technical arguments, demonstrating that our technical approach and model assumptions were reasonable and provide a conservatively high estimate of the potential for reproductive success when compared to observed nest inundation events. We conclude with a description of the realities faced by the Platte River Recovery Implementation Program (PRRIP) as we integrate learning to adjust management actions.

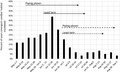

**Linked Article**: https://doi.org/10.1002/ece3.4109

## 1 INTRODUCTION

We appreciate the opportunity to respond to Alexander, Jorgensen, and Bomberger‐Brown's (hereafter AJB's) letter to the editor of Ecology and Evolution (Alexander et al. 2018). We begin by restating the principal findings of our study to correct AJBs’ mischaracterization of our work beginning in the abstract where they attribute to us a principal assertion that “interior least tern and piping plovers are not adapted to occupying and nesting on river sandbars on the Platte River system.” We made no such assertion. These species do occupy Platte River sandbars. Our research focused on the potential for on‐channel reproductive success in the contemporary lower Platte River (LPR) and historical and contemporary central Platte River (CPR) finding that: (1) there is no evidence that interior least terns (*Sternula antillarum athalassos*; hereafter, least tern) and piping plovers (*Charadrius melodus*) are physiologically adapted to begin nesting concurrent with the recession of spring floods in the Platte River basin; (2) there are many years when no successful on‐channel reproduction is possible because emergent sandbar habitat is inundated after most nests have been initiated; (3) the limited potential for reproductive success thus limits the potential for maintenance of stable subpopulations via on‐channel nesting habitat alone; and (4) the availability and use of off‐channel habitats, like sandpits, may have allowed for these species to develop stable subpopulations in a river basin where hydrology is not ideally suited to their nesting ecology.

The remainder of this response addresses AJB's major points of criticism under their topic subheadings in the order that the subjects were addressed in our original manuscript. In instances where we have included figures or tables that expand upon our emergent sandbar habitat model results, we focus on the contemporary LPR segment as it is the segment with the highest potential for reproductive success and is cited by AJB as an example of a resilient and dynamic natural system (along with the historical AHR) that benefits these species.

## 2 THE HISTORICAL RECORD

AJB assert that we “overlooked portions of the historical record which demonstrate terns and plovers were regularly present and successfully nested along the central Platte River (CPR) and lower Platte River (LPR).” They support this assertion by summarizing early historical references to the occurrence of least terns and piping plovers in Nebraska. Anecdotal observations such as the Bruner, Wolcott, and Swenk (1904) assessment that least terns were “not a rare breeder” in Nebraska are neither evidence for or against AJB's assertion that the species successfully nested along the CPR and LPR. Neither do they speak to the purpose of our study, which was to evaluate the reproductive ecology of these species in relation to historical and contemporary AHR and contemporary LPR hydrology and sandbar dynamics.

Simply put, the first observation of on‐channel least tern nesting in the AHR occurred in 1942 when a colony was discovered nesting on the river near Lexington, Nebraska by Dr. Ray S. Wycoff (Wycoff, 1960). That colony was observed nesting on a low sandbar in the channel, a high in‐channel island created by sand mining, and at adjacent sandpits. The first observations of piping plovers in the AHR are more general in nature, but indicate that some on‐channel nesting may have occurred in the early 1950s (Pitts, 1988). The first observation of least tern and piping plover nesting in the LPR occurred in 1941 when both species were observed nesting on a sandbar near Columbus (Ducey, 1985).

These observations occurred near the end of large‐scale surface water development in the Platte Basin when the channel was actively adjusting to hydrologic alteration (Murphy, Randle, Fotherby, & Daraio, 2004; Simons & Associates Inc. and URS Greiner Woodward Clyde, 2000, Williams, 1978). The various authors (Currier, Lingle, & VanDerwalker, 1985; National Research Council 2005, USFWS 2006) that concluded the AHR supported populations of both species prior to water development inferred a decline in species use and productivity from (1) the reduction in AHR channel width from the predevelopment period, (2) a reduction in the magnitude of the spring rise resulting in unsuitably low sandbar habitat likely to be inundated during the nesting season, (3) a lack of on‐channel nesting in the contemporary AHR, and (4) species use of the contemporary LPR. This inference assumes physical conditions in the historical AHR were similar to the contemporary LPR and the LPR currently supports viable species subpopulations. We examined the first assumption in Section 4 of our original manuscript, finding that the potential for successful nesting in the historical AHR was likely much lower than the contemporary LPR due to important differences in‐channel width and discharge magnitude. The second assumption is addressed in following sections of this response.

In their discussion of the historical records, AJB also state the presence of species populations on other Great Plains rivers like the Niobrara that lack off‐channel habitats provide additional evidence “contradicting the notion that adjacent that off‐channel habitats are a prerequisite for these species to colonize and breed within a river segment.” We consider this a straw man argument (Talisse & Aikin, 2006). Our findings were specific to the Platte River study segments we evaluated, and we did not generalize to other segments or river systems. We concur with AJB that the Niobrara supports stable species subpopulations in the absence of off‐channel habitats (Adolf, Higgins, Kruse, & Pavelka, 2001). As such, it provides a valuable contrast to the AHR that has been explored by the PRRIP as part of a larger peer‐reviewed data synthesis project (PRRIP 2015, Chapter 6).

## 3 COMPARISONS OF PLATTE RIVER HYDROGRAPH WITH NEST INITIATION DATE DISTRIBUTIONS

In this portion of their critique, AJB criticizes our comparison of species nest initiation periods to the annual hydrograph of the historical and contemporary Platte. They conclude that it would have been more informative to plot the timing and magnitude of instantaneous annual peak discharges in relation to nesting periods. AJB's focus on the instantaneous annual peak discharge assumes that it is the only discharge relevant to species reproductive potential. This is a flawed assumption. As discussed in our study, AJB's critique, and in subsequent section of this response, sandbars do not build to the peak stage of formative events making them vulnerable to inundation at discharges lower than the instantaneous annual peak. Consequently, the timing of the instantaneous annual peak does not speak to the presence or absence of habitat‐inundating flow events during the species’ nesting periods. Our emergent sandbar habitat model was developed to explicitly assess the frequency and timing of such events in relation to species nesting periods.

**Figure 1:**
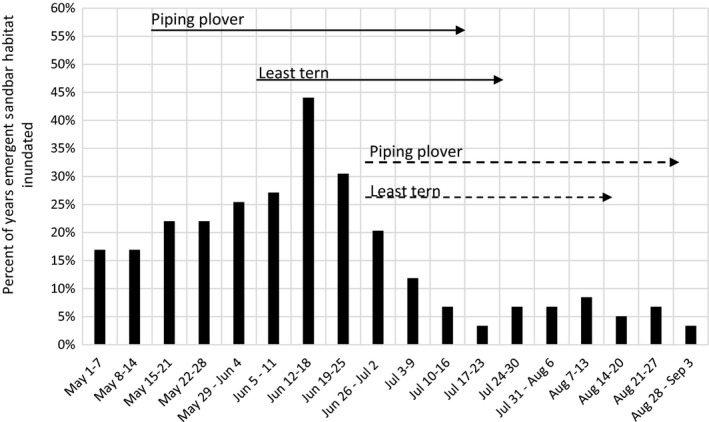
Weekly sandbar model results indicating the percent of years when sandbar habitat in the contemporary LPR was inundated along with the period necessary for successful nesting and brood rearing for each species. Bars indicate the percent of years when sandbar habitat was inundated for one or more days during that week. Solid lines represent periods necessary for successful nesting beginning at peak nest initiation dates. Dashed lines represent periods necessary for successful nesting following mid‐June inundation events

Emergent sandbar habitat model results for the contemporary LPR Reach are presented in Figure 1 along with the period necessary for successful nesting and brood rearing for each species. We also present a summary of annual inundation events as well as the number of days sandbar habitat was inundated (Table 1). Model results indicate that sandbar habitat is inundated at least one time during the nesting season (1‐May to 30‐August) in most years with a median duration of 6 days. Inundation occurs most frequently in June with the highest potential for inundation in mid‐June (44% of years; Figure 1).

**Table 1:** Emergent sandbar habitat model results for the LPR Segment including the number of annual habitat inundation events during the nesting period (1 May to 30 August) and total habitat inundation duration in days

	5th	25th	Median	75th	95th
Number of inundation events	0	1	1	2	3
Inundation duration (days)	0	1	6	14	34

As illustrated in Figure 1 and Table 1, LPR emergent sandbar habitat is inundated in >75% of years during the nesting period (1‐May to 30‐Aug) with the highest proportion of inundation events occurring during the latter half of June. Due to the greater availability of emergent sandbar habitat in the early portion of the nesting period, both species often initiate many nests prior to inundating events in mid‐ to late June resulting in high levels of renesting in early to mid‐July. In order for these species to routinely avoid June inundation events, they would need to begin initiating nests in either early April to fledge prior to mid‐June or begin initiating nests in early July after the June peak. We are unaware of any evidence from any regional river system indicating that this is currently or has ever been the case.

From a subpopulation viability perspective, we have found reproductive success of AHR nests initiated late in the breeding season (mid‐July) is often lower due to fewer eggs typically being laid in a clutch and can further be reduced if not initiated in time to successfully fledge chicks (DMB, pers. obs.). Our sandbar habitat model did not assess differences in productivity throughout the nesting season as there is little information on the success of late renesting on sandbar habitat. Additional systematic monitoring of late renesting on sandbars would allow for a more thorough assessment of this issue.

## 4 DISTRIBUTIONS OF NEST INITIATION DATES AND ASSOCIATED NESTING PERIODS

AJB correctly note that our analysis of nest initiation dates only includes data from the AHR (2001–2013) and nearly all the nest initiation dates come from off‐channel habitats. As indicated in our manuscript, development of on‐channel nesting periods was not possible as there are very few on‐channel nest records in the AHR and there is no systematic season‐long monitoring of on‐channel habitat in the LPR. AJB state that use of nest initiation data from static, human‐created, off‐channel habitat is an incomplete representation of species breeding phenology which could easily result in incorrect or misleading conclusions when applied to species’ behavior in dynamic river systems. We too shared this concern.

In our study, we assessed the appropriateness of our nesting periods by comparing them to the range of nest initiation dates reported in the LPR (Brown & Jorgensen, 2008, 2009, 2010; Brown, Jorgensen, & Dinan, 2011, 2012, 2013) to identify any disparities. Ninety percent of reported LPR nest initiation dates fell within the 90% nesting periods we developed using AHR data (Brown & Jorgensen, 2008, 2009, 2010; Brown et al., 2011, 2012, 2013; Farnsworth, Baasch, Smith, & Werbylo, 2017; PRRIP 2015). AJB did not dispute this finding.

The only additional information for the LPR segment is found in Kirsch (1996). Kirsch compared least tern nesting dates (1987–1990) and found no difference in nesting periods for on‐channel and off‐channel habitats. Kirsch did, however, note that there was more late nesting and renesting on river habitat than on sandpits due to nest inundation. This is also consistent with the findings of our study.

## 5 FORMATIVE RIVER STAGE, EMERGENT SANDBAR HEIGHT, AND NESTING HEIGHT

AJBs’ critique of our emergent sandbar habitat model focuses on four main issues: (1) the use of primarily off‐channel nest initiation dates to develop the least tern and piping plover nesting periods; (2) the lack of a detailed description of sandbar height data collection and analysis methods; (3) the model assumption of a constant maximum sandbar height in relation to peak stage of habitat‐forming flow events (AJB refer to this as the stage gap); and (4) the model assumption that species nests occur at mean sandbar height. The use of off‐channel nest initiation dates has been discussed in the previous section titled “Distributions of Nest Initiation Dates and Associated Nesting Periods.” Each of the remaining critiques will be addressed in turn followed by a discussion of model performance.

### 5.1 Sandbar height data collection and analysis methods description

Given the range of disciplines addressed in our manuscript (i.e., hydrology, hydraulics, sandbar dynamics, and species nesting ecology), our focus on the emergent sandbar model, and the target audience of this journal, we chose to simplify the methods section of the manuscript. An expanded description of the method used to evaluate sandbar heights in the AHR can be found in PRRIP (2015). We refer readers to that document.

### 5.2 Sandbar height model parameter values

AJB’s critique in this area focuses on our assumption of a constant sandbar height (stage gap) for all habitat‐forming peak flow events, which we defined as the maximum mean daily peak discharge occurring during a 1.5‐year period ending on 1 July of the current model year. AJB state that our use of a single value ignores evidence suggesting a pattern of increasing stage gap with increasing discharge. AJB provide two lines of evidence. The first is in the form of several studies (Brice, 1964; Cant & Walker, 1978; Mohrig & Smith, 1996; Smith, 1971) that, as AJB state, “indicate that sandbars submerged during low‐magnitude discharges often have shallow gaps at their crests.” AJB link this general observation to the stage gap for habitat‐forming peak flow events by hypothesizing that there is a Froude limit to vertical sandbar growth that results in an increasing stage gap with increasing discharge. This hypothesis is logical but untested. Accordingly, we have no way to address the veracity of this component of the critique or assess the potential magnitude of a Froude effect in relation to the many other factors that influence sandbar height, including bed material grain size (Ikeda, 1984), sediment supply (Germanoski & Schumm, 1993), and event duration (Crowley, 1981).

AJBs’ second line of evidence is related to the findings of Alexander, Schultze, and Zelt (2013). AJB indicate that LPR sandbars surveyed in the spring of 2011 were created during a large 2010 high flow event and sandbars surveyed in the summer and fall of 2011 were created during a smaller 2011 peak flow event that occurred after the spring bar survey. AJB then cite a smaller stage gap for summer/fall 2011 surveys as evidence that the stage gap is smaller for lower‐magnitude events. We refer readers to figure 8 of Alexander et al. (2013), which includes peak flow stages and sandbar frequency distributions. Depending on the gage that is referenced, between 10% and 40% of the summer and fall 2011 bar height frequency distributions exceed 2011 peak stage.

Summer and fall 2011 bar area exceeding 2011 peak stage could not have been created during the 2011 peak flow event instead figure 8 demonstrates they likely represent portions of 2010 bars that persisted through the 2011 event. For these surveys to be used as evidence for a smaller stage gap at lower discharges, the data would need to be parsed to remove bar area that that persisted from 2010. For this reason, we solely used Alexander et al.'s (2013) spring bar height distribution to develop our LPR sandbar height model parameter estimate, with the caveat that we related the median (not mean as stated by AJB) height from the distribution to the stage associated with the mean daily peak flow (as opposed to instantaneous peak) to be consistent with other model input parameters.

We have also analyzed AHR sandbar heights following four peak flow events ranging from 190 to 434 m^3^/s in magnitude (2‐year to 13‐year return interval) with event durations ranging from 33 to 98 days (Table 2). We did not observe an increase in stage gap with increasing discharge. Instead, median sandbar height appears to increase slightly with increasing event duration, although median heights are not statistically different from one another.

**Table 2:** Results of AHR sandbar height analyses during the period of 2010–2015

	2010	2011	2014	2015
Event duration (days)	54	98	33	77
Even volume (millions of cm)	566	1,541	261	1,594
Peak date at Kearney Gage	6/17/2010	6/25/2011	6/14/2014	6/18/2015
Mean peak discharge for AHR (m^3^/s)	233	251	190	434
Median sandbar height below peak (m)	0.45	0.38	0.5	0.44
Standard deviation of sandbar height below peak (m)	0.14	0.18	0.18	0.18

### 5.3 Model assumption of nesting at median sandbar height

In this portion of their critique, AJB cite tables from Ziewitz, Sidle, and Dinan (1992) and tables/figures from three other publications (Alexander et al., 2013; Brown & Jorgensen, 2008; Smith & Renken, 1991) as empirical evidence to support statements that (1) sandbars with nests tend to have mean elevations that are higher than unoccupied bars and (2) nest heights tend to be located on higher regions of a sandbar's topography. AJB then conclude that since least terns and piping plovers select higher sandbars and nest in higher locations on those sandbars, our model certainly underestimates the potential for successful nesting. We note that Ziewitz et al. (1992) reported mean and maximum sandbar heights at used and systematic sites in the AHR and LPR were not significantly different. Likewise, Brown and Jorgensen (2008) reported mean and maximum sandbar height for used and unused LPR bars in their analysis were not statistically different. Despite the lack of a statistical difference in bar height at used and unused sites, these species may indeed tend to nest on higher bars and/or higher regions of a sandbar's topography. Comparisons of observed inundation events with emergent sandbar model results provide a straightforward way to assess AJB's conclusion that our model, therefore, underestimates the potential for successful nesting.

### 5.4 Emergent sandbar model performance

In our original manuscript (Section 3.3), we assessed model performance through the comparison of observed instances of on‐channel nest inundation in the historical and contemporary AHR and contemporary LPR to model predictions for those events. For the purposes of our response, we have expanded these comparisons to encompass LPR inundation events during 1989–1990 (Kirsch, 1996; Sidle, Carlson, Kirsch, & Dinan, 1992) as well as nesting and inundation events during the period of 2008–2017 (Brown & Jorgensen, 2008, 2009, 2010; Brown, Jorgensen, & Dinan, 2014, 2015, 2016, 2017; Brown et al., 2011, 2012, 2013). Comparison results are presented in Table 3.

**Table 3:** Comparisons of observed on‐channel habitat conditions and nesting in the lower Platte River in relation to Farnsworth et al. (2017) emergent sandbar habitat model predictions

Year	Reported habitat inundation	Model inundation	Piping plover nesting observations	Piping Plover model success window	Least tern nesting observations	Least tern model success window
1989	High flows on 28 June inundated many sandbars	Bars inundated on 28 June to 2 July	18 nests, 7 flooded, no hatch or fledge info	No potential	85 nests, 21 inundated, 0.57 fledglings per pair	10 days
1990	Sandbars inundated 14 June to 19 June	Bars inundated 16 June to 20 June and on 26 July	28 nests flooded, 13 renests, 6 flooded, no hatch or fledge info.	No potential	94 nests inundated, 93 renests, 0.25 fledglings per pair	No potential
2008	High flows mid‐May through mid‐June. Bar habitat available after mid‐June	Bars inundated 28 May through 13 June	3 nests, 1 hatched (July 24). No fledging info.	10 days	150 nests, 63 confirmed/likely hatched, no fledging info.	29 days
2009	Peak flow 21 June completely inundated many sandbars	No inundation	47 nests, 14 inundated, 12 confirmed/likely hatched. No fledging info.	Season‐long	264 nests, 50 inundated, 110 confirmed/likely hatched. No fledging info.	Season‐long
2010	Large mid‐June peak flow inundated sandbars	Bars inundated 11‐June to 24‐June	8 nests prior to flooding, none after	No potential	5 nests prior to flooding, Four colonies after. No fledging info.	18 days
2011	Bars inundated in late May and again in late June	No inundation	10 nests, 7 inundated in May, 3 confirmed/likely hatched. No fledging info.	Season‐long	98 nests, 56 inundated, 38 confirmed/likely successful. No fledging info.	Season‐long
2012	No inundation observed	Bars inundated on 30 May	4 nests, no hatch info.	24 Days	74 nests, no hatch info.	43 days
2013	Many bars at least partially inundated in late May	Bars inundated 27 May to 12 June.	11 nests, 4 likely hatched. No fledging info.	No potential	53 nests, 9 likely hatched. No fledging info.	15 days
2014	Bars inundated until mid‐July	Bars inundated intermittently 12 May to 4 July	No nests	No potential	26 nests, no hatch or fledge info.	4 days
2015	Bars inundated May to mid‐July	Bars inundated intermittently 7 May to 3 July	No nests	No potential	8 nests, no hatch or fledge info.	9 days
2016	Majority bars inundated to mid‐June	Bars inundated intermittently 20 April to 22 June	No nests	No potential	33 nests, no hatch or fledge info.	5 days
2017	Most sandbars inundated until early to mid‐June	Bars inundated intermittently 1 May to 27 May	3 nests. No hatching info.	18 days	70 nests, 16 chicks, 13 fledglings	32 days

Our model is consistently conservative in that it slightly under‐predicts the potential for and length of inundation when compared to observed inundation events (Table 3). This is largely due to our decision to use mean daily discharge values in sandbar inundation calculations. During high flow events, daily instantaneous peak discharge is often substantially higher than mean daily discharge. A comparison of annual instantaneous peak and mean daily peak discharges for the period of 1954–2016 provides an indication of the magnitude of differences (Table 4). During this period, 50% of instantaneous peak discharges were more than 238 m^3^/s greater than the mean daily peak discharge, which equates to a 0.13 m difference in peak stage. Put another way, our model underestimates the maximum stage associated with instantaneous peak discharges by more than 0.13 m in 50% of years. As a result, our model necessarily under‐predicts the potential for nest inundation on any given day.

**Table 4:** Difference in instantaneous and mean daily discharge and stage in the lower Platte River

	Difference in discharge (m^3^/s)	Difference in stage (m)	Difference in stage (in)
5th percentile	58	0.04	1.69
25th percentile	120	0.08	3.00
Median	238	0.13	5.16
75th percentile	388	0.17	6.66
95th percentile	924	0.37	14.58

## 6 LEAST TERN AND PIPING PLOVER POPULATION ECOLOGY

This portion of AJBs’ critique asserts that the fledge ratio‐based assessment of the potential for long‐term maintenance of stable, on‐channel species subpopulations (no off‐channel habitat) described in our Discussion Section was too simple to address complex questions about metapopulation dynamics. We respond to this criticism by demonstrating that our simple assessment leads to the same inference as the recent Catlin et al. (2016) piping plover metapopulation study that included the LPR segment.

Our model predicted that there was no potential for piping plover reproductive success in 42% of years in the contemporary LPR. The long‐term average fledge ratio target proposed to be necessary in the Platte basin to maintain a stable piping plover population is 1.13 fledglings per breeding pair (Lutey, 2002). Therefore, average piping plover productivity in years with some potential for reproductive success would need to exceed 1.95 fledglings per breeding pair (1.13/0.58) to achieve the fledge ratio target of 1.13 over the long term. We noted that we are not aware of any habitat that supports this level of average reproductive success leading us to the conclusion that it is unlikely that LPR on‐channel habitat alone can support a stable piping plover subpopulation.

Catlin et al. (2016) examined three piping plover subpopulations on the lower Platte and Missouri Rivers during the period of 2008–2013, including the evaluation of habitat loss and renewal due to natural peak flow events. Model results indicated a low probability of metapopulation extinction over 100 years. However, the persistence of the lower Platte River subpopulation as well as the metapopulation were reported to be dependent on static off‐channel habitat that provided a stable source of nesting habitat through time. This conclusion is consistent with our assessment that in‐channel habitat in the contemporary LPR is not capable of sustaining a stable subpopulation of piping plovers and that off‐channel habitats provide the stable source of habitat necessary to do so.

We are not aware of the existence of a similar metapopulation study for least terns, but would note that there appears to be greater potential for the maintenance of a stable, on‐channel subpopulation in the LPR segment as the average fledge ratio estimate (0.84 fledglings per pair) to achieve the Lutey (2002) objective over the long term has at least been periodically reported on LPR on‐channel habitats (Brown & Jorgensen, 2008, 2009).

## 7 MANAGEMENT AND POLICY IMPLICATIONS

In this section of their critique, AJB argue that the creation and maintenance of off‐channel nesting habitat in the contemporary AHR is an inferior alternative to on‐channel habitat that could be created through some form of river restoration that would eliminate the need for human intervention. This is a direct appeal to nature (Moore & Baldwin, 1993) which assumes, without supporting evidence, that restoration of historical AHR channel morphology and hydrology would produce sandbar habitat with a high potential for reproductive success. Our emergent sandbar habitat model for the historical AHR, which utilizes historical hydrology and channel morphology, indicates very limited potential for least tern or piping plover reproductive success.

AJB also cite the contemporary LPR as an example of a resilient and dynamic river system that benefits these species, inferring that it is a restoration example for the AHR. This ignores the reality of the similarities in the magnitude of off‐channel nesting in both the AHR and LPR. In the AHR, approximately 96% of nests initiated since 2001 have occurred on off‐channel habitats. Likewise, in the contemporary LPR, a plurality of nests are initiated on off‐channel habitats. Since 2008, approximately 90% of reported LPR piping plover nests and 70% of reported LPR tern nests have been initiated on off‐channel habitat (Brown & Jorgensen, 2008, 2009, 2010; Brown et al., 2011, 2012, 2013, 2014, 2015, 2016, 2017).

From an implementation perspective, AJB also ignore the reality of socioeconomic and resources constraints. The Platte River is one of the most highly developed river systems in the world with 9 billion m^3^ of reservoir storage distributed across multiple large irrigation and flood control reservoirs (Murphy et al., 2004, Simons and Associates Inc. & URS Greiner Woodward Clyde 2000). The PRRIP is a collaborative endangered species recovery program (PRRIP 2006a) tasked with providing defined benefits to these species while still providing for necessary agricultural and municipal water uses in the Platte River basin including the domestic water supply for millions of people in the Denver metropolitan area (PRRIP 2006a).

The PRRIP utilizes adaptive management to reduce uncertainty regarding key scientific and technical uncertainties and aid decision‐making (Compass Resource Management, Inc. 2016; PRRIP 2006b). In relation to least terns and piping plovers, the PRRIP invested nearly a decade in implementation of large‐scale adaptive management experiments to test the effectiveness of on‐ and off‐channel habitat creation and management strategies. Once those experiments were completed, the PRRIP conducted a formal structured decision‐making process and fully evaluated trade‐offs and consequences of various on‐ and off‐channel habitat management strategies. This process resulted in a decision to adjust actions for least terns and piping plovers in a manner that incorporates a combination of off‐channel habitat, on‐channel habitat, and flow management guidance (Compass Resource Management, Inc. 2016).

## CONFLICT OF INTEREST

None declared.

## AUTHORS' CONTRIBUTION

All authors contributed in all phases of the development of our response to the letter to the editor as well as all analyses of data contained therein.
